# PERSON-CENTERED CARE RELATED TO RESOURCE USE, RESIDENT QUALITY OF LIFE, AND STAFF JOB STRAIN IN SWEDISH NURSING HOMES

**DOI:** 10.1093/geroni/igz038.141

**Published:** 2019-11-08

**Authors:** Annica Backman, Anders Sköldunger

**Affiliations:** Department of Nursing Umea University, Umea, Sweden

## Abstract

A critical challenge facing aged care systems throughout the world is to meet the complex care needs of a growing population of older persons. Although person-centred care has been advocated as the “gold standard” and a key component of high quality of care, the significance of care utilization in person-centred units as well as the impact of person-centred care on resident quality of life and staff job strain in nursing home care is yet to be explored. Thus, the aim was to explore person-centred care and its association to resource use, resident quality of life and staff job strain. The study is based on a cross-sectional national survey and data on 4831 residents and 3605 staff were collected by staff in 2014, deriving from nursing homes in 35 Swedish municipalities. In this study, descriptive statistics and regression modelling were used to explore this association. The preliminary results showed that person-centred care was positively associated to resource use (i.e care hours) and resident quality of life in Swedish nursing homes, when controlling for resident age, gender and cognitive status. Person-centred care was negatively associated to staff perception of job strain. This indicates that person-centred care provision seem to increase resource use (i.e. slightly more care hours utilized) but also beneficially impact resident quality of life as well as alleviate care burden in terms job strain among staff.

